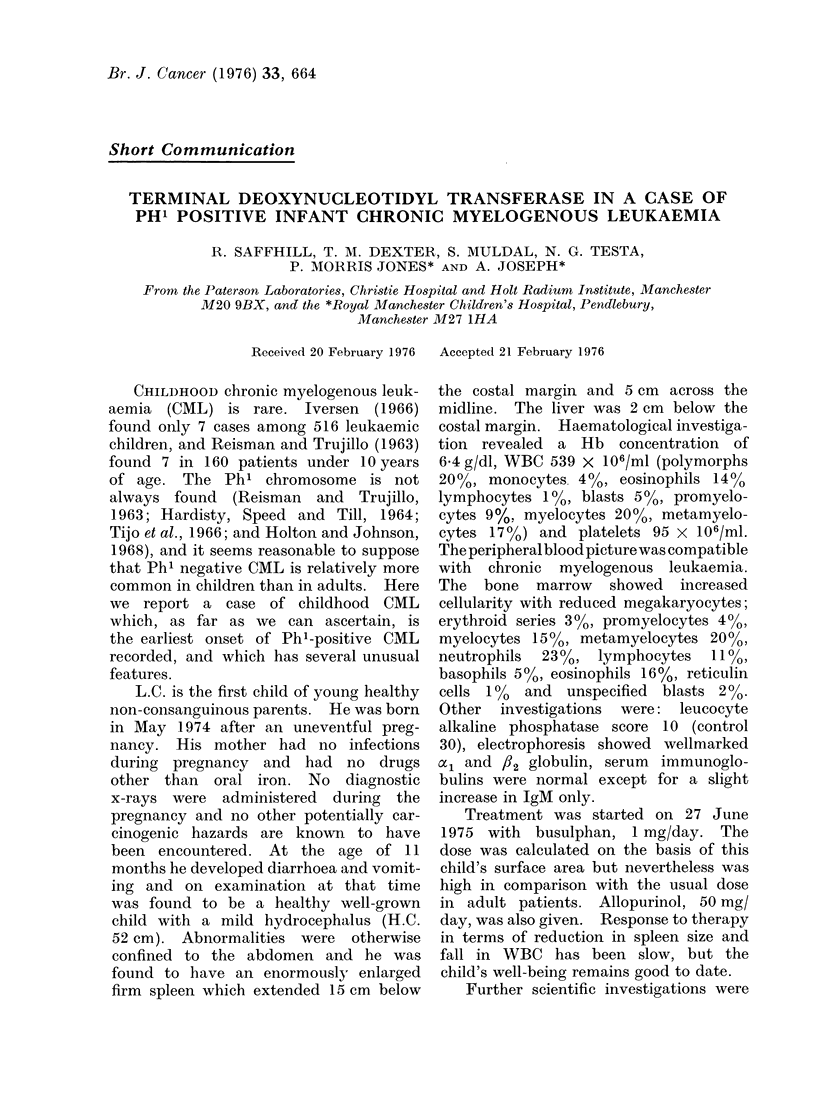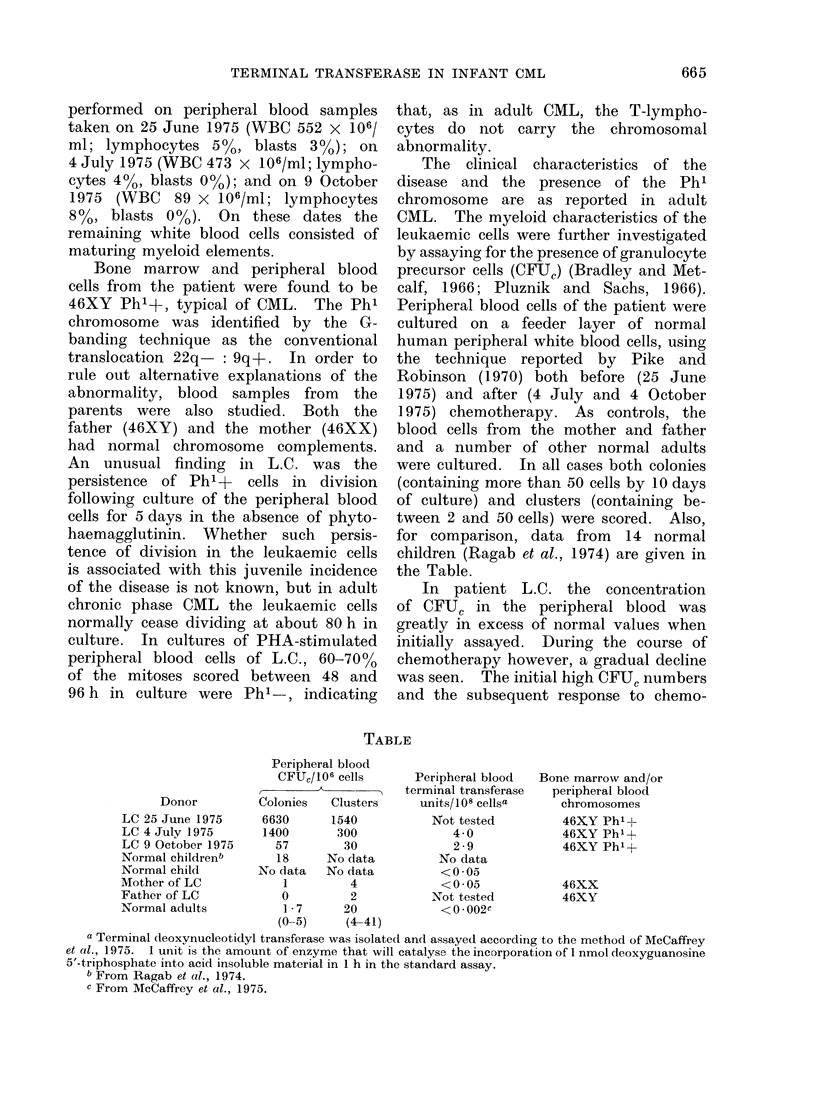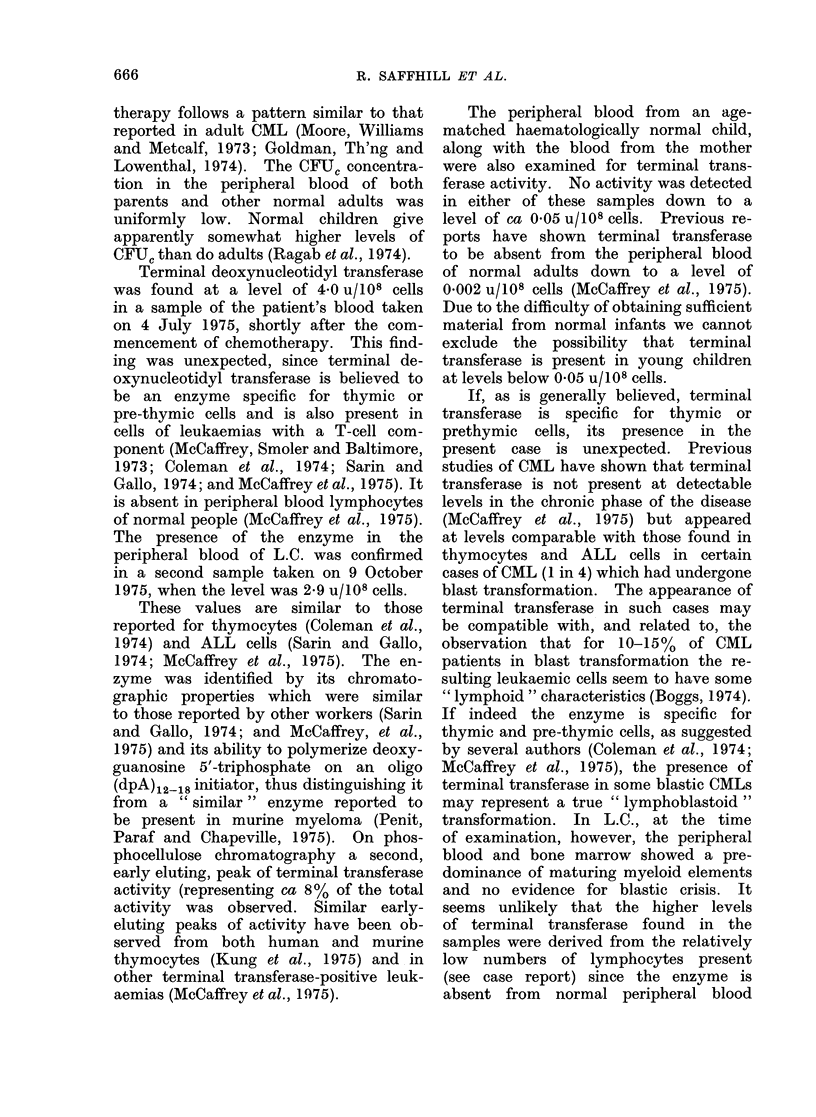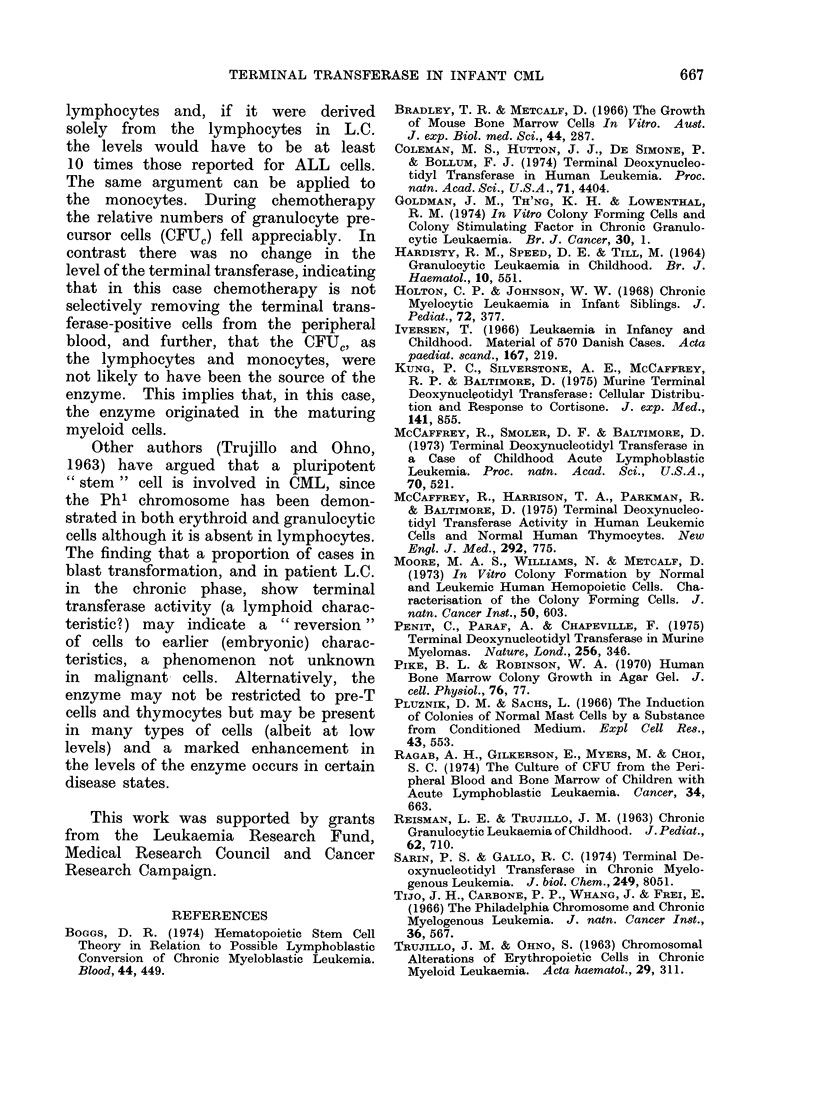# Terminal deoxynucleotidyl transferase in a case of PH positive infant chronic myelogenous leukamia.

**DOI:** 10.1038/bjc.1976.105

**Published:** 1976-06

**Authors:** R. Saffhill, T. M. Dexter, S. Muldal, N. G. Testa, P. M. Jones, A. Joseph


*Br. J. Cancer* (1976) **33**, 664

***Short Communication***

### TERMINAL DEOXYNUCLEOTIDYL TRANSFERASE IN A CASE OF PH[1] POSITIVE INFANT CHRONIC MYELOGENOUS LEUKAEMIA

R. SAFFHILL, T. M. DEXTER, S. MULDAL, N. G. TESTA, P. MORRIS JONES* AND A. JOSEPH*

*From the Paterson Laboratories, Christie Hospital and Holt Radium Institute, Manchester M20 9BX, and the *Royal Manchester Children's Hospital, Pendlebury, Manchester M27 1HA*

Received 20 February 1976 Accepted 21 February 1976

CHILDHOOD chronic myelogenous leukaemia (CML) is rare. Iversen (1966) found only 7 cases among 516 leukaemic children, and Reisman and Trujillo (1963) found 7 in 160 patients under 10 years of age. The Ph[1] chromosome is not always found (Reisman and Trujillo, 1963; Hardisty, Speed and Till, 1964; Tijo *et al.*, 1966; and Holton and Johnson, 1968), and it seems reasonable to suppose that Ph[1] negative CML is relatively more common in children than in adults. Here we report a case of childhood CML which, as far as we can ascertain, is the earliest onset of Ph[1]-positive CML recorded, and which has several unusual features.

L.C. is the first child of young healthy non-consanguinous parents. He was born in May 1974 after an uneventful pregnancy. His mother had no infections during pregnancy and had no drugs other than oral iron. No diagnostic x-rays were administered during the pregnancy and no other potentially carcinogenic hazards are known to have been encountered. At the age of 11 months he developed diarrhoea and vomiting and on examination at that time was found to be a healthy well-grown child with a mild hydrocephalus (H.C. 52 cm). Abnormalities were otherwise confined to the abdomen and he was found to have an enormously enlarged firm spleen which extended 15 cm below the costal margin and 5 cm across the midline. The liver was 2 cm below the costal margin. Haematological investigation revealed a Hb concentration of 6·4 g/dl, WBC 539 $\times$ $10^6$/ml (polymorphs 20%, monocytes 4%, eosinophils 14% lymphocytes 1%, blasts 5%, promyelocytes 9%, myelocytes 20%, metamyelocytes 17%) and platelets 95 $\times$ $10^6$/ml. The peripheral blood picture was compatible with chronic myelogenous leukaemia. The bone marrow showed increased cellularity with reduced megakaryocytes; erythroid series 3%, promyelocytes 4%, myelocytes 15%, metamyelocytes 20%, neutrophils 23%, lymphocytes 11%, basophils 5%, eosinophils 16%, reticulin cells 1% and unspecified blasts 2%. Other investigations were: leucocyte alkaline phosphatase score 10 (control 30), electrophoresis showed wellmarked $\alpha_1$ and $\beta_2$ globulin, serum immunoglobulins were normal except for a slight increase in IgM only.

Treatment was started on 27 June 1975 with busulphan, 1 mg/day. The dose was calculated on the basis of this child's surface area but nevertheless was high in comparison with the usual dose in adult patients. Allopurinol, 50 mg/day, was also given. Response to therapy in terms of reduction in spleen size and fall in WBC has been slow, but the child's well-being remains good to date.

Further scientific investigations were performed on peripheral blood samples taken on 25 June 1975 (WBC 552 × $10^6$/ml; lymphocytes 5%, blasts 3%); on 4 July 1975 (WBC 473 × $10^6$/ml; lymphocytes 4%, blasts 0%); and on 9 October 1975 (WBC 89 × $10^6$/ml; lymphocytes 8%, blasts 0%). On these dates the remaining white blood cells consisted of maturing myeloid elements.

Bone marrow and peripheral blood cells from the patient were found to be 46XY $Ph^1$+, typical of CML. The $Ph^1$ chromosome was identified by the G-banding technique as the conventional translocation 22q− : 9q+. In order to rule out alternative explanations of the abnormality, blood samples from the parents were also studied. Both the father (46XY) and the mother (46XX) had normal chromosome complements. An unusual finding in L.C. was the persistence of $Ph^1$+ cells in division following culture of the peripheral blood cells for 5 days in the absence of phytohaemagglutinin. Whether such persistence of division in the leukaemic cells is associated with this juvenile incidence of the disease is not known, but in adult chronic phase CML the leukaemic cells normally cease dividing at about 80 h in culture. In cultures of PHA-stimulated peripheral blood cells of L.C., 60–70% of the mitoses scored between 48 and 96 h in culture were $Ph^1$−, indicating that, as in adult CML, the T-lymphocytes do not carry the chromosomal abnormality.

The clinical characteristics of the disease and the presence of the $Ph^1$ chromosome are as reported in adult CML. The myeloid characteristics of the leukaemic cells were further investigated by assaying for the presence of granulocyte precursor cells ($CFU_c$) (Bradley and Metcalf, 1966; Pluznik and Sachs, 1966). Peripheral blood cells of the patient were cultured on a feeder layer of normal human peripheral white blood cells, using the technique reported by Pike and Robinson (1970) both before (25 June 1975) and after (4 July and 4 October 1975) chemotherapy. As controls, the blood cells from the mother and father and a number of other normal adults were cultured. In all cases both colonies (containing more than 50 cells by 10 days of culture) and clusters (containing between 2 and 50 cells) were scored. Also, for comparison, data from 14 normal children (Ragab *et al.*, 1974) are given in the Table.

In patient L.C. the concentration of $CFU_c$ in the peripheral blood was greatly in excess of normal values when initially assayed. During the course of chemotherapy however, a gradual decline was seen. The initial high $CFU_c$ numbers and the subsequent response to chemo-

TABLE

| Donor | Peripheral blood $CFU_c/10^6$ cells: Colonies | Clusters | Peripheral blood terminal transferase units/$10^8$ cells[a] | Bone marrow and/or peripheral blood chromosomes |
|---|---|---|---|---|
| LC 25 June 1975 | 6630 | 1540 | Not tested | 46XY $Ph^1$+ |
| LC 4 July 1975 | 1400 | 300 | 4·0 | 46XY $Ph^1$+ |
| LC 9 October 1975 | 57 | 30 | 2·9 | 46XY $Ph^1$+ |
| Normal children[b] | 18 | No data | No data | |
| Normal child | No data | No data | <0·05 | |
| Mother of LC | 1 | 4 | <0·05 | 46XX |
| Father of LC | 0 | 2 | Not tested | 46XY |
| Normal adults | 1·7 (0–5) | 20 (4–41) | <0·002[c] | |

[a] Terminal deoxynucleotidyl transferase was isolated and assayed according to the method of McCaffrey *et al.*, 1975. 1 unit is the amount of enzyme that will catalyse the incorporation of 1 nmol deoxyguanosine 5′-triphosphate into acid insoluble material in 1 h in the standard assay.
[b] From Ragab *et al.*, 1974.
[c] From McCaffrey *et al.*, 1975.

therapy follows a pattern similar to that reported in adult CML (Moore, Williams and Metcalf, 1973; Goldman, Th'ng and Lowenthal, 1974). The $CFU_c$ concentration in the peripheral blood of both parents and other normal adults was uniformly low. Normal children give apparently somewhat higher levels of $CFU_c$ than do adults (Ragab *et al.*, 1974).

Terminal deoxynucleotidyl transferase was found at a level of 4·0 u/$10^8$ cells in a sample of the patient's blood taken on 4 July 1975, shortly after the commencement of chemotherapy. This finding was unexpected, since terminal deoxynucleotidyl transferase is believed to be an enzyme specific for thymic or pre-thymic cells and is also present in cells of leukaemias with a T-cell component (McCaffrey, Smoler and Baltimore, 1973; Coleman *et al.*, 1974; Sarin and Gallo, 1974; and McCaffrey *et al.*, 1975). It is absent in peripheral blood lymphocytes of normal people (McCaffrey *et al.*, 1975). The presence of the enzyme in the peripheral blood of L.C. was confirmed in a second sample taken on 9 October 1975, when the level was 2·9 u/$10^8$ cells.

These values are similar to those reported for thymocytes (Coleman *et al.*, 1974) and ALL cells (Sarin and Gallo, 1974; McCaffrey *et al.*, 1975). The enzyme was identified by its chromatographic properties which were similar to those reported by other workers (Sarin and Gallo, 1974; and McCaffrey, *et al.*, 1975) and its ability to polymerize deoxyguanosine 5′-triphosphate on an oligo $(dpA)_{12-18}$ initiator, thus distinguishing it from a " similar " enzyme reported to be present in murine myeloma (Penit, Paraf and Chapeville, 1975). On phosphocellulose chromatography a second, early eluting, peak of terminal transferase activity (representing *ca* 8% of the total activity was observed. Similar early-eluting peaks of activity have been observed from both human and murine thymocytes (Kung *et al.*, 1975) and in other terminal transferase-positive leukaemias (McCaffrey *et al.*, 1975).

The peripheral blood from an age-matched haematologically normal child, along with the blood from the mother were also examined for terminal transferase activity. No activity was detected in either of these samples down to a level of *ca* 0·05 u/$10^8$ cells. Previous reports have shown terminal transferase to be absent from the peripheral blood of normal adults down to a level of 0·002 u/$10^8$ cells (McCaffrey *et al.*, 1975). Due to the difficulty of obtaining sufficient material from normal infants we cannot exclude the possibility that terminal transferase is present in young children at levels below 0·05 u/$10^8$ cells.

If, as is generally believed, terminal transferase is specific for thymic or prethymic cells, its presence in the present case is unexpected. Previous studies of CML have shown that terminal transferase is not present at detectable levels in the chronic phase of the disease (McCaffrey *et al.*, 1975) but appeared at levels comparable with those found in thymocytes and ALL cells in certain cases of CML (1 in 4) which had undergone blast transformation. The appearance of terminal transferase in such cases may be compatible with, and related to, the observation that for 10–15% of CML patients in blast transformation the resulting leukaemic cells seem to have some " lymphoid " characteristics (Boggs, 1974). If indeed the enzyme is specific for thymic and pre-thymic cells, as suggested by several authors (Coleman *et al.*, 1974; McCaffrey *et al.*, 1975), the presence of terminal transferase in some blastic CMLs may represent a true " lymphoblastoid " transformation. In L.C., at the time of examination, however, the peripheral blood and bone marrow showed a predominance of maturing myeloid elements and no evidence for blastic crisis. It seems unlikely that the higher levels of terminal transferase found in the samples were derived from the relatively low numbers of lymphocytes present (see case report) since the enzyme is absent from normal peripheral blood lymphocytes and, if it were derived solely from the lymphocytes in L.C. the levels would have to be at least 10 times those reported for ALL cells. The same argument can be applied to the monocytes. During chemotherapy the relative numbers of granulocyte precursor cells ($CFU_c$) fell appreciably. In contrast there was no change in the level of the terminal transferase, indicating that in this case chemotherapy is not selectively removing the terminal transferase-positive cells from the peripheral blood, and further, that the $CFU_c$, as the lymphocytes and monocytes, were not likely to have been the source of the enzyme. This implies that, in this case, the enzyme originated in the maturing myeloid cells.

Other authors (Trujillo and Ohno, 1963) have argued that a pluripotent "stem" cell is involved in CML, since the $Ph^1$ chromosome has been demonstrated in both erythroid and granulocytic cells although it is absent in lymphocytes. The finding that a proportion of cases in blast transformation, and in patient L.C. in the chronic phase, show terminal transferase activity (a lymphoid characteristic?) may indicate a "reversion" of cells to earlier (embryonic) characteristics, a phenomenon not unknown in malignant cells. Alternatively, the enzyme may not be restricted to pre-T cells and thymocytes but may be present in many types of cells (albeit at low levels) and a marked enhancement in the levels of the enzyme occurs in certain disease states.

This work was supported by grants from the Leukaemia Research Fund, Medical Research Council and Cancer Research Campaign.

### REFERENCES

BOGGS, D. R. (1974) Hematopoietic Stem Cell Theory in Relation to Possible Lymphoblastic Conversion of Chronic Myeloblastic Leukemia. *Blood*, **44**, 449.

BRADLEY, T. R. & METCALF, D. (1966) The Growth of Mouse Bone Marrow Cells *In Vitro*. *Aust. J. exp. Biol. med. Sci.*, **44**, 287.

COLEMAN, M. S., HUTTON, J. J., DE SIMONE, P. & BOLLUM, F. J. (1974) Terminal Deoxynucleotidyl Transferase in Human Leukemia. *Proc. natn. Acad. Sci., U.S.A.*, **71**, 4404.

GOLDMAN, J. M., TH'NG, K. H. & LOWENTHAL, R. M. (1974) *In Vitro* Colony Forming Cells and Colony Stimulating Factor in Chronic Granulocytic Leukaemia. *Br. J. Cancer*, **30**, 1.

HARDISTY, R. M., SPEED, D. E. & TILL, M. (1964) Granulocytic Leukaemia in Childhood. *Br. J. Haematol.*, **10**, 551.

HOLTON, C. P. & JOHNSON, W. W. (1968) Chronic Myelocytic Leukaemia in Infant Siblings. *J. Pediat.*, **72**, 377.

IVERSEN, T. (1966) Leukaemia in Infancy and Childhood. Material of 570 Danish Cases. *Acta paediat. scand.*, **167**, 219.

KUNG, P. C., SILVERSTONE, A. E., MCCAFFREY, R. P. & BALTIMORE, D. (1975) Murine Terminal Deoxynucleotidyl Transferase: Cellular Distribution and Response to Cortisone. *J. exp. Med.*, **141**, 855.

MCCAFFREY, R., SMOLER, D. F. & BALTIMORE, D. (1973) Terminal Deoxynucleotidyl Transferase in a Case of Childhood Acute Lymphoblastic Leukemia. *Proc. natn. Acad. Sci., U.S.A.*, **70**, 521.

MCCAFFREY, R., HARRISON, T. A., PARKMAN, R. & BALTIMORE, D. (1975) Terminal Deoxynucleotidyl Transferase Activity in Human Leukemic Cells and Normal Human Thymocytes. *New Engl. J. Med.*, **292**, 775.

MOORE, M. A. S., WILLIAMS, N. & METCALF, D. (1973) *In Vitro* Colony Formation by Normal and Leukemic Human Hemopoietic Cells. Characterisation of the Colony Forming Cells. *J. natn. Cancer Inst.*, **50**, 603.

PENIT, C., PARAF, A. & CHAPEVILLE, F. (1975) Terminal Deoxynucleotidyl Transferase in Murine Myelomas. *Nature, Lond.*, **256**, 346.

PIKE, B. L. & ROBINSON, W. A. (1970) Human Bone Marrow Colony Growth in Agar Gel. *J. cell. Physiol.*, **76**, 77.

PLUZNIK, D. M. & SACHS, L. (1966) The Induction of Colonies of Normal Mast Cells by a Substance from Conditioned Medium. *Expl Cell Res.*, **43**, 553.

RAGAB, A. H., GILKERSON, E., MYERS, M. & CHOI, S. C. (1974) The Culture of CFU from the Peripheral Blood and Bone Marrow of Children with Acute Lymphoblastic Leukaemia. *Cancer*, **34**, 663.

REISMAN, L. E. & TRUJILLO, J. M. (1963) Chronic Granulocytic Leukaemia of Childhood. *J. Pediat.*, **62**, 710.

SARIN, P. S. & GALLO, R. C. (1974) Terminal Deoxynucleotidyl Transferase in Chronic Myelogenous Leukemia. *J. biol. Chem.*, **249**, 8051.

TIJO, J. H., CARBONE, P. P., WHANG, J. & FREI, E. (1966) The Philadelphia Chromosome and Chronic Myelogenous Leukemia. *J. natn. Cancer Inst.*, **36**, 567.

TRUJILLO, J. M. & OHNO, S. (1963) Chromosomal Alterations of Erythropoietic Cells in Chronic Myeloid Leukaemia. *Acta haematol.*, **29**, 311.